# Solid‐State Fluorescent Carbon Dots with Unprecedented Efficiency from Visible to Near‐Infrared Region

**DOI:** 10.1002/advs.202205788

**Published:** 2022-12-03

**Authors:** Bin Xu, Jie Li, Jing Zhang, Huiying Ning, Xiaoqi Fang, Jian Shen, Heng Zhou, Tianlong Jiang, Zhenhua Gao, Xiangeng Meng, Zifei Wang

**Affiliations:** ^1^ School of Materials Science & Engineering Qilu University of Technology (Shandong Academy of Sciences) Jinan 250353 China

**Keywords:** carbon dots, high quantum yields, near‐infrared, solid‐state fluorescence, white LED

## Abstract

Developing solid‐state luminescent materials with bright long‐wavelength emissions is of considerable practical importance in light‐emitting diodes (LEDs) but remains a formidable challenge. Here, a novel structure engineering strategy is reported to realize solid‐state fluorescence (FL)‐emitted carbon dots (CDs) from visible to near‐infrared region. This is the first report of such an extended wavelength emission of self‐quenching‐resistant solid‐state CDs. Notably, the quantum yields of these CDs are remarkably improved up to 67.7%, which is the highest value for solid‐state CDs. The surface polymer chains of CDs can efficiently suppress the conjugated sp^2^ carbon cores from *π*–*π* stacking inducing aggregation caused FL quenching, and the redshift of FL emissions is attributed to narrowing bandgap caused by an enlarged sp^2^ carbon core. Using these CDs as conversion phosphors, the fabrication of white LEDs with adjustable correlated color temperatures of 1882–5019 K is achieved. Moreover, a plant growth LED device is assembled with a blue‐LED chip and deep‐red/near‐infrared‐emitted CDs. Compared with sunlight and white LEDs, the peanuts irradiated by plant growth LED lamp show higher growth efficiency in terms of branches and leaves. This work provides high‐quality solid‐state CD‐based phosphors for LED lighting sources that are required for diverse optoelectronic applications.

## Graphical abstract

This work realizes solid‐state fluorescence‐emitted carbon dots (CDs) from the visible to near‐infrared region. The quantum yields of these CDs are remarkably improved up to 67.7%, which is the highest value for solid‐state CDs without matrix. Both white light‐emitting diodes (LEDs) with correlated color temperatures of 1882–5019 K and plant growth LED lamp are fabricated.

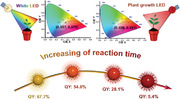

## 1 Introduction

Carbon dots (CDs), as an emerging class of luminescent nanoparticles consisting of carbonaceous structures with various functional groups, have received much attention in optical devices due to their excellent luminescence properties, use of abundant and eco‐friendly precursors, simple preparation without harsh reaction conditions, and low toxicity.^[^ ^1^, ^2^, ^3^, ^4^, ^5^ ^]^ Until now, the tremendous advance has been achieved in the synthesis of CDs’ solution that exhibits a photoluminescence (PL) quantum yield (QY) of ≈90% from blue to red emission.^[^ ^6^, ^7^, ^8^, ^21^ ^]^ To realize the real lighting device applications of CDs, fabrication of solid‐state fluorescence (FL)‐emitted CDs is requisite. Nevertheless, CDs constantly undergo serious self‐quenching in the solid state due to excessive resonance energy transfer or direct *π*–*π* interactions resulting from the van der Waals interaction between adjacent layers.^[^ ^9^, ^10^ ^]^

To circumvent the drawback, a routine method is to incorporate the CDs into the appropriate host matrices such as organic polymer, starch, inorganic salts, and silica xerogel.^[^ ^11^, ^12^, ^13^, ^14^, ^15^ ^]^ However, the mixing path always has the disadvantage of poor photostability and uneven dispersion, as well as the problems of low QYs. Therefore, it is essential to search for a rational strategy to obtain self‐quenching‐resistant solid‐state FL characteristics by efficiently restraining the *π*–*π* stacking interaction and excessive Förster resonance energy transfer (FRET) of CDs in bulk or powder form without the addition of external substances. Several groups have been targeted to develop efficient self‐quenching‐resistant solid‐state CDs by a structural design strategy.^[^ ^16^, ^17^, ^18^, ^19^, ^20^ ^]^ Recently, we also reported red/green/blue solid‐state FL carbon quantum rings with high QYs up to 30–46%,^[^ ^9^ ^]^ achieved by linking adjacent curved carbon quantum ribbons of different lengths. However, efficient long‐wavelength emissive CDs, especially the deep‐red and near‐infrared (NIR) regions (>640 nm), are very scarce thanks to lack of rational solutions and fuzzy luminescence mechanisms. Moreover, the QYs of self‐quenching‐resistant solid‐state CDs are usually less than 30%, except for highly efficient blue/green CDs, which dramatically hinder their practical application in the solid‐state lighting device. Therefore, it is still highly desirable to develop an effective method for synthesizing solid‐state long‐wavelength emissive CDs with high QYs.

In this study, we report a novel structure engineering strategy to synthesize highly luminescent CDs, having remarkably tunable self‐quenching‐resistant solid‐state FL emissions from visible (Vis, 570 nm) to NIR (721 nm) region. The relative QYs of these CDs reached 67.7–5.4%, which are the highest values for solid‐state CDs without matrices. Detailed structural characterizations and elaborate molecular dynamics (MD) calculations reveal that the abundant surface polymer chains of CDs can prevent the carbon cores from the *π*–*π* stacking effect in the aggregation state, and the redshift of solid‐state FL emissions is ascribed to the increased conjugated sp^2^ carbon core. By employing these CDs as phosphors, we have achieved the fabrication of three white light‐emitting diodes (LEDs) with adjustable correlated color temperatures (CCTs) of 1882–5019 K at a 20 mA drive current. In addition, a plant growth LED lamp combined with deep‐red‐ and NIR‐emitted CDs, and a blue‐LED chip is also fabricated. The PL spectrum and the absorption bands of plant photosynthetic pigments are well matched in blue and red regions. Compared with sunlight and white LEDs, the plants irradiated by a growth LED lamp show better growth in terms of leaves and rhizome length under the same experimental conditions.

## 2 Results and Discussion

**Figure 1:**
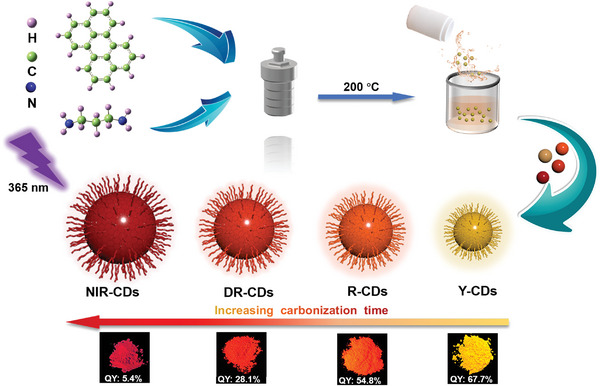
Schematic illustration of the fabrication process of CDs with tunable color from yellow (570 nm) to NIR (721 nm), and optical images of Y‐CDs, R‐CDs, DR‐CDs, and NIR‐CDs under 365 nm UV light.

**Figure 2:**
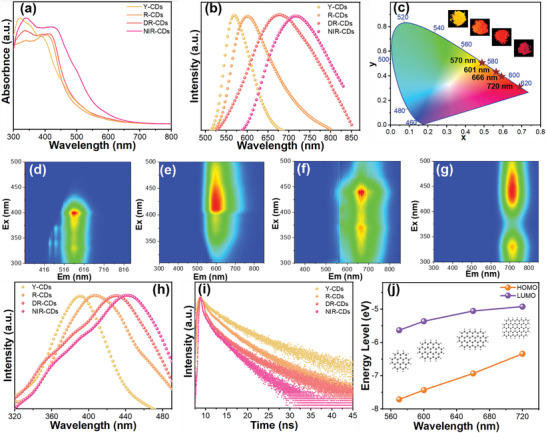
a) UV–vis absorption, b) Normalized FL emission spectra, c) CIE coordinate diagram of FL emissions of Y‐, R‐, DR‐, and NIR‐CDs, respectively. d–g) 2D contour map FL spectra of Y‐, R‐, DR‐, and NIR‐CDs, respectively. h) Normalized excitation spectra and i) time‐resolved FL spectra of Y‐, R‐, DR‐, and NIR‐CDs, respectively. j) Dependence of the HOMO and LUMO energy levels on the FL emissions of Y‐, R‐, DR‐, and NIR‐CDs.

The synthesis of solid‐state FL emissive CDs from the Vis to NIR regions (named as yellow emitted CDs (Y‐CDs), red emitted CDs (R‐CDs), deep‐red emitted CDs (DR‐CDs), and NIR‐CDs, respectively) involved the solvothermal treatment of perylene, 1,3‐diaminopropane, and sulfuric acid at 200 °C for different reaction times (**Figure** **1**; see the details in the “Experimental Section” of the Supporting Information). The sulfuric acid reagent is chosen as a catalyst, which easily promotes the carbonization process during the reaction. The obtained Y‐, R‐, DR‐, and NIR‐CD powders show bright yellow, red, deep‐red, and NIR lights with QYs of 67.7–5.4% under ultraviolet (UV) lamp (365 nm) irradiation (Figure 1; Figures S1 and S2, Supporting Information). To the best of our knowledge, this is a record value for self‐quenching‐resistant solid‐state CDs (Table S1, Supporting Information). As shown in **Figure** **2**a, the four CDs show similar UV–vis absorption peaks at 300–370 nm, which can be assigned to the n–π* transitions of CDs. The UV–vis spectra of these CDs exhibit strong excitonic absorption peaks centered at 394 nm (Y‐CDs), 412 nm (R‐CDs), 422 nm (DR‐CDs), and 445 nm (NIR‐CDs), respectively, indicating the formation of conjugated sp^2^ carbon cores in these CDs. The normalized FL spectra of these CDs also display emission peaks at about 570 nm (Y‐CDs), 604 nm (R‐CDs), 666 nm (DR‐CDs), and 721 nm (NIR‐CDs), respectively (Figure 2b). The emission color of the FL is quantitatively characterized by the Commission International de I'Eclairage (CIE) color coordinates, clearly displaying a profound color transformation from yellow to NIR (Figure 2c). As presented in Figure 2d–g, 2D contour FL spectra show that the FL of Y‐, R‐, DR‐, and NIR‐CDs can be excited with a wide range of excitation wavelengths, and the maximum emission peaks are independent of the excitation wavelengths. The optimal excitation peaks of Y‐, R‐, DR‐, and NIR‐CDs are centered at ≈391, ≈408, ≈431, and ≈446 nm, respectively (Figure 2h), which agree well with their corresponding excitonic absorption peak wavelengths, indicating that these FL emissions are relative to band edge exciton states.^[^ ^21^ ^]^ The FL decay curves can be fitted by a double‐exponential function, with average lifetimes estimated as 2.35, 1.03, 0.92, and 0.68 ns for Y‐, R‐, DR‐, and NIR‐CDs, respectively (Figure 2i; Table S2, Supporting Information). The optical properties of CDs’ aqueous dispersion are also characterized in Figures S3–S7 (Supporting Information). The characteristics of absorption and emission spectra are similar to those of solid‐state CDs, which further proves the bandgap luminescence. The slight decay (<9.6%) is measured for Y‐, R‐, DR‐, and NIR‐CDs, respectively (Figure S8, Supporting Information). When the temperature reached 160 ℃, the maximum emission wavelengths of FL spectra showed a slightly change, and the FL intensity remained as high as 87.1%, 92.9%, 95.3%, 94.1％ of their initial value at 20 ℃ (Figures S9 and S10, Supporting Information). The results indicated high thermal stability of Y‐R‐, DR‐, and NIR‐CDs over a temperature range of 20‐160℃，comparable to that of rare‐earth based phosphors.^[^ ^17^, ^22^ ^]^ Bandgap energies of the Y‐, R‐, DR‐, and NIR‐CDs are calculated by the equation *E*_g_^opt^  =  1240/*λ*_edge_ (*λ*_edge_ is the onset value of the first excitonic absorption peaks in the direction of longer wavelengths).^[^ ^23^, ^24^ ^]^ The *E*_g_^opt^  gradually decreased from 2.18 to 1.92 eV with the transition of FL emission from 570 to 721 nm, further certifying the quantum size effect of CDs.^[^ ^2^ ^]^ Otherwise, the highest occupied molecular orbital (HOMO) energy level is calculated by UV photoelectron spectroscopy (Figure S11, Supporting Information). The end edge of 4.55 eV (Y‐CDs), 4.21 eV (R‐CDs), 5.03 eV (DR‐CDs), and 5.23 eV (NIR‐CDs) can be estimated from the cross point of *X* = 0 and bevel edge tangent. Then, the values of the HOMO energy levels of Y‐, R‐, DR‐, and NIR‐CDs can be figured out to be −7.71, −7.43, −6.93, and −6.34 eV (*E*_HOMO_ = − (21.22 − (*E*_End edge_ − *E*_Fermi_))). Therefore, the values of lowest unoccupied molecular orbital (LUMO) energy level can be calculated to be −5.63, −5.36, −5.05, and −4.62 eV for Y‐, R‐, DR‐, and NIR‐CDs, respectively (*E*_LUMO_ = *E*_HOMO_ + *E*_g_^opt^). The decrease of HOMO energy levels and increase of the LUMO energy levels directly reveal the bandgap transitions in these CDs (Figure 2j).

**Figure 3:**
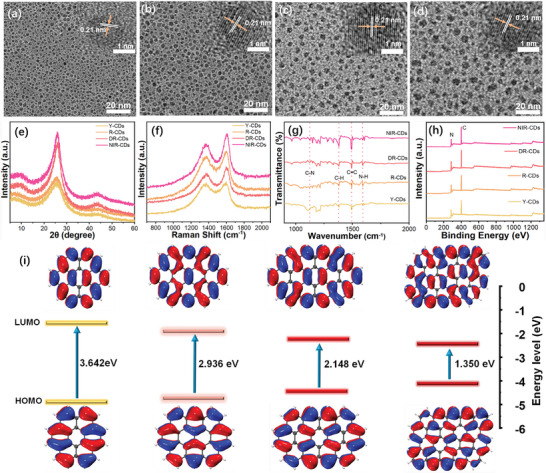
a–d) TEM images of Y‐, R‐, DR‐, and NIR‐CDs. The inset is the HRTEM images of Y‐, R‐, DR‐, and NIR‐CDs. e) XRD pattern, f) Raman spectra, g) XPS spectra, and h) FT‐IR spectra of Y‐, R‐, DR‐, and NIR‐CDs, respectively. i) HOMO and LUMO states of the established model by increasing the aromatic rings.

Transmission electron microscopy (TEM) images show the Y‐, R‐, DR‐, and NIR‐CDs to be well dispersed without aggregation and exhibit a spherical particle shape (**Figure** **3**a–d). Their average lateral sizes are about ≈1.9, 2.3, 3.1, and 3.5 nm for Y‐, R‐, DR‐, and NIR‐CDs (Figure S12, Supporting Information), respectively. As shown in the inset images in Figure 3a–d, the high‐resolution TEM (HRTEM) pictures show a high crystallinity with an in‐plane lattice spacing of 0.21 nm, which corresponds to (100) planes of graphite carbon.^[^ ^25^ ^]^ The atomic force microscopy (AFM) images show Y‐, R‐, DR‐, and NIR‐CDs with average topographic heights of ≈1.75, 2.48, 2.83, and 3.53 nm (Figure S13, Supporting Information), respectively, in agreement with the TEM characterization. The results indicate that the redshift of FL from Y‐CDs to NIR‐CDs could be ascribed to quantum size effects. The X‐ray diffraction (XRD) patterns reveal the distinct characteristic peak at around 25.6°, which is attributed to an interlayer spacing of 0.34 nm (Figure 3e). The increase in the intensity of the distinct peak in the XRD patterns demonstrates that the degree of graphitization increases from Y‐CDs to NIR‐CDs.^[^ ^26^ ^]^ The high degree of graphitization of CDs is further reflected in Raman spectra (Figure 3f). The intensity ratios (*I*_D_/*I*_G_) of the disordered D band at 1370 cm^−1^ and crystalline G band at 1604 cm^−1^ are 0.98, 0.92, 0.89, and 0.86 from Y‐CDs to NIR‐CDs, respectively. The increasing ratio values imply the strengthened graphitization degree from Y‐CDs to NIR‐CDs, suggesting a gradual increase size of sp^2^ carbon cores, which is consistent with the high crystallinity degree of these CDs.^[^ ^27^ ^]^ The chemical compositions of the Y‐, R‐, DR‐, and NIR‐CDs are investigated by Fourier transform‐infrared spectroscopy (FT‐IR) and X‐ray photoelectron spectroscopy (XPS). The FT‐IR spectra show that these CDs contain C—N (1126 cm^−1^), C—H (1381 cm^−1^), C=C (1493 cm^−1^), and N—H (1592 cm^−1^) functional groups or chemical bonds (Figure 3g).^[^ ^28^ ^]^ All the full XPS spectra show two strong peaks at 284.5 and 400.8 eV (Figure 3h), which are ascribed to C 1s and N 1s, respectively.^[^ ^29^ ^]^ The content of C gradually increases, and the contents of N decrease from Y‐CDs to NIR‐CDs further suggest the increase in the degree of sp^2^ carbon cores, which are consistent with their increased particle size and crystalline degree of these CDs as determined by TEM measurements (Tables S3 and S4, Supporting Information). The C 1s band exhibits two peaks at 284.3 eV for C—C/C=C and 285.2 eV for C—N (Figure S14, Supporting Information).^[^ ^30^, ^31^ ^]^ As presented in Figure S15 (Supporting Information), the N 1s spectra reveal the presence of C—N (399.6 eV) and N—H (400.8 eV).^[^ ^32^ ^]^

**Figure 4:**
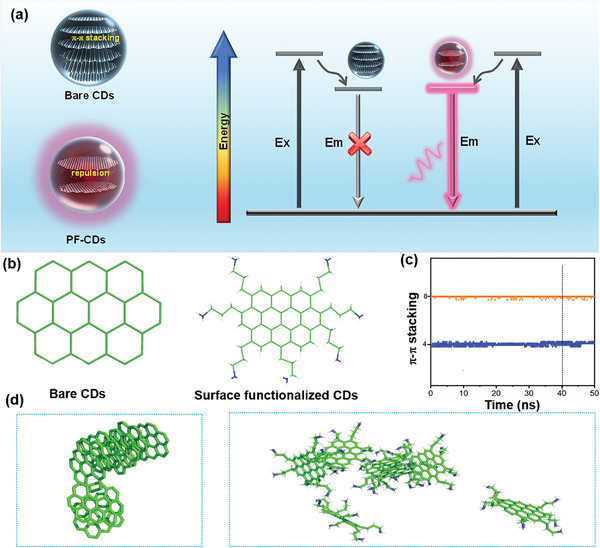
a) Schematic illustration and energy level diagram of self‐quenching‐resistant FL of PF‐CDs. b) Bare CDs and PF‐CDs wherein the ground state (S_0_) were optimized by B3LYP/6‐31G** at the density functional theory level. c) *π*–*π* stacking counts over the MD simulation time (50 ns) for bare CDs and PF‐CDs. d) Snapshots at 40.3 ns of bare CDs and PF‐CDs in a bulk amorphous system (*N* = 7 molecules).

Based on structural and spectroscopic characterizations, it is evident that these CDs constitute a sp^2^ carbon core with a high degree of crystallinity with the surface of abundant polymer chains, yielding the strong solid‐state FL emission. One possible mechanism of structure formation is as follows: 1) we think that precursor of CDs is crucial for synthesizing the efficient Y‐, R‐, DR‐, and NIR‐CDs. Single perylene could be considered as the smallest sp^2^ carbon domain, which acts as a building block to form a sp^2^ carbon crystal cluster, while surface functionalization can be achieved by chemically bonding in the edge of the CDs; 2) the Y‐, R‐, DR‐, and NIR‐CDs are then synthesized through controlled polymerization, deamination carbonization reaction of perylene, and 1,3‐diaminopropane by changing the reaction time and adding sulfuric acid as a catalyst and a dehydrating agent. The FL emissions of these CDs originate from band edge exciton‐state decay, and redshift FL emission is attributed to narrowing bandgap caused by an enlarged sp^2^ carbon crystal cluster. This speculation is also verified by theoretical calculation (Figure 3i; Figure S16, Supporting Information). The surfaces of the CDs retained plentiful polymer chains, which can control the interparticle distance, thereby weakening the direct *π*–*π* interaction and excessive FRET, and achieve self‐quenching‐resistant solid‐state FL characteristics (**Figure** **4**a). The polymer chains can also be supported by the viscosity characteristics of the CDs’ aqueous dispersion. The viscosity decreases with the increase of temperature, which accords with the typical characteristics of polymers (Figure S17, Supporting Information).

To further prove the experimental results, we conduct MD incorporating quantum‐chemical calculation simulations for bare CDs and polymer‐chain‐functionalized CD (PF‐CD) models by setting periodic boundary conditions to count the *π*–*π* stacking events in the thermodynamic equilibrium state.^[^ ^18^ ^]^ Figure 4b and Figure S18 (Supporting Information) show the simplified molecular structures proposed to represent the bare CDs and PF‐CDs (ten‐membered benzene rings) used in the experiment. Figure 4c shows the calculated number of *π*–*π* stacking events for the bare CD and PF‐CD models of ≈50 ns duration, respectively. It clearly shows that the count of the *π*–*π* stacking of PF‐CDs at 40.3 s (far after this onset of convergence) is smaller than that of bare CDs. Figure 4d shows the corresponding snapshots of the simulated molecular arrangements of the bare CDs and PF‐CDs obtained at 40.3 ns. This tendency in the corresponding snapshots is matching with the *π*–*π* stacking event count. In conclusion, the PF‐CDs could increase the distance between adjacent PF‐CDs in the solid state, and weakens the direct *π*–*π* interaction and excessive FRET between them, thus emitting solid‐state FL.

**Figure 5:**
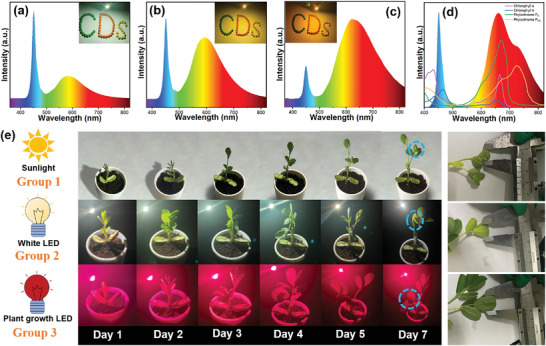
a–c) PL emission spectra of the white LEDs based on Y‐CDs, Y‐/R‐CDs, and Y‐/R‐/DR‐CDs, respectively (inset, the object color under prepared white LEDs). d) PL spectrum of the plant growth LED device based on DR‐/NIR‐CDs and absorption spectra of chlorophyll A and B and phytochrome P_R_ and P_FR_. e) Panoramic images of peanut growth for 7 days under the condition of sunlight, white LED, and plant growth LED.

Given the CDs’ multicolored solid‐state emission, ultrahigh QY, and high thermostability, the Y‐, R‐, DR‐CDs synthesized herein can be used to make white LEDs with tunable CCTs. As a preliminary demonstration, the Y‐CDs are applied to prepare CDs/epoxy composite, then coated on the blue‐LED (450 nm) chip, followed by curing at 60 °C for 45 min to form a white LED. **Figure** **5**a and Figure S19 (Supporting Information) show the PL spectrum of the as‐fabricated white LED (white LED‐1) with a CCT of 5019 K, CIE color coordinates of (0.357, 0.373), a color rendering index (CRI) of 75.9, and a luminous efficiency of 54.6 lm W^−1^ under an operating current of 20 mA, which is comparable to that of some rare‐earth‐based white LED (Table S5, Supporting Information). As we all know, the warm white LED with low CCT (CCT < 4000 K) is strongly desired for indoor lighting because its comfortable ambient lighting that can stave off eye fatigue.^[^ ^33^, ^34^ ^]^ In consequence, two warm white LEDs (white LED‐2 and white LED‐3) with CCTs of 2285 and 1882 K are fabricated by employing mixtures with Y‐/R‐CDs and Y‐/R‐/DR‐CDs, respectively. PL spectra of the as‐fabricated warm white LEDs are shown in Figure 5b,c, which also cover the whole visible light region from 400 to 800 nm, similar to that of their powders. The CIE color coordinates, luminous efficiency, and CRI of these warm white LEDs are (0.497, 0.416) and (0.520, 0.389), 35.8, and 24.3 lm W^−1^, and 82.4 and 87.5, respectively (Figure S20, Supporting Information). To test the stability of these white LED devices, the PL spectra of three white LEDs were measured against working time at 20 mA drive current. As shown in Figure S21 (Supporting Information), the PL spectra have insignificant change after three white LEDs worked 15 h, demonstrating a good stability.

Recently, the applications of horticultural lighting have attracted increasing interest in plant growth. In particular, the blue (400–500 nm), red (600–700 nm), and far‐red (700–780 nm) absorption bands play a vital role in the development of plant photosynthetic pigments (chlorophyll A, chlorophyll B, phytochrome P_R_, and phytochrome P_FR_) for plants.^[^ ^35^ ^]^ Thus, it is of considerable importance to develop a plant growth LED device with bright deep‐red emissions for indoor plant cultivation. A plant growth LED light is fabricated by directly employing the mixture of DR‐CDs and NIR‐CDs as phosphors (Figure S22, Supporting Information). The PL spectrum of the manufactured LED device is mainly composed of the blue band (420–500 nm) from to blue‐LED chip and the red band (570–800 nm) from DR‐/NIR‐CDs, as presented in Figure 5d, which matches the absorption wavelength of plant pigment (chlorophyll A and B, and phytochrome P_R_ and P_FR_), indicates that DR‐CDs and NIR‐CDs can be a candidate for the plant growth LED lamp. Hence, the as‐fabricated LED as a plant growth lighting source is assessed. Peanut is employed as a model plant, and peanut cultivation under the same conditions is carried out except for the light source. The samples are divided into three groups according to the different light sources (group 1: sunlight, group 2: as‐prepared white LED‐1, and group 3: plant growth LED). According to the photos of peanut growth in Figure 5e and Figure S23 (Supporting Information), it can be clearly seen that the plant growth LED device can effectively improve the overall photosynthesis reaction of peanuts, and the peanuts grown under plant growth LED lighting have higher growth efficiency with more robust branches and leaves than peanuts under sunlight and white LED‐1. After 7 days, the average leaf area value is measured from three groups with a Vernier caliper (Figure 5e). The result shows that the cotyledons of the peanut in group 3 are more prominent, and the leaves are greener (Figure S23, Supporting Information). The fresh weight and height of plant are also analyzed in Figures S24 and S25 (Supporting Information). Similarly, the fresh weight and height of plants in the group 3 are significantly higher than the groups 1 and 2. All the results imply that this plant growth LED can effectively promote photosynthesis reaction and improve the growth of plant cotyledons.

## 3 Conclusion

In conclusion, we demonstrated a structure engineering method to manipulate highly efficient solid‐state FL emissive CDs from the Vis to NIR region. The QYs of these CDs are remarkably improved up to 67.7%, which is higher than that of previously reported solid‐state CDs. The synthesis strategy relies on the reasonable selection of precursors and introduced acid reagents in the carbonization process, which plays a role in generating color‐tunable solid‐state PL phenomena of as‐produced CDs. The experiments and molecular dynamics revealed that the abundant surface polymer chains of CDs can efficiently suppress the conjugated sp^2^ carbon cores from the *π*–*π* stacking effect in the solid state. Moreover, the redshift solid‐state PL emission is attributed to a narrowing bandgap caused by an enlarged sp^2^ carbon crystal cluster, illustrating the quantum size effect of as‐prepared CDs. The advantages of the CDs in ultrahigh QYs, color tunability, remarkable photostability, and thermal stability are perfectly integrated to construct high‐performance white LED architectures. In detail, three white LEDs are fabricated by coating Y‐CDs, mixtures with Y‐/R‐CDs, and combinations with Y‐/R‐/DR‐CDs on a blue‐LED chip, which show favorable white light characteristics with CCTs from 5019 to 1882 K, CRI from 75.9 to 87.5, the CIE color coordinates shift from (0.357, 0.373) to (0.520, 0.389), and luminous efficiency from 54.6 to 24.3 lm W^−1^. Eventually, an LED device was assembled with a blue‐LED chip and the as‐synthesized DR‐/NIR‐CDs’ phosphor that emits bright red light, which matches the plant absorption spectra well. The LED device can be used as a plant growth light source to grow peanut plants. Under the same experimental conditions, the peanuts irradiated by plant LED lamps showed higher growth efficiency in terms of branches and leaves. Therefore, this work shows that our synthetic CD phosphors are promising for the design of a wide range of LED devices, and hope to have a broader application in other fields.

## 4 Experimental Section

Materials and experimental details are included in the Supporting Information.

## Conflict of Interest

The authors declare no conflict of interest.

## Supporting information

Supporting Information

## Data Availability

Research data are not shared.

## References

[1] H. Wu , W. Su , H. Xu , Y. Zhang , Y. Li , X. Li , L. Fan , View 2021, 2, 20200061.

[2] L. Wang , W. Li , L. Yin , Y. Liu , H. Guo , J. Lai , Y. Han , G. Li , M. Li , J. Zhang , R. Vajtai , P. M. Ajayan , M. Wu , Sci. Adv. 2020, 6, eabb6772. 33008913 10.1126/sciadv.abb6772 PMC7852397

[3] Y. Shi , H. Xu , T. Yuan , T. Meng , H. Wu , J. Chang , H. Wang , X. Song , Y. Li , X. Li , Y. Zhang , W. Xie , L. Fan , Aggregate 2021, 3, e108.

[4] H. Choi , S. J. Ko , Y. Choi , P. Joo , T. Kim , B. R. Lee , J. W. Jung , H. J. Choi , M. Cha , J. R. Jeong , I. W. Hwang , M. H. Song , B. S. Kim , J. Y. Kim , Nat. Photonics 2013, 7, 732.

[5] F. Yuan , Y. Wang , G. Sharma , Y. Dong , X. Zheng , P. Li , A. Johnston , G. Bappi , J. Fan , H. Kung , B. Chen , M. I. Saidaminov , K. Singh , O. Voznyy , O. M. Bakr , Z. Lu , E. H. Sargent , Nat. Photonics 2019, 14, 171.

[6] X. Wang , Y. Ma , Q. Wu , Z. Wang , Y. Tao , Y. Zhao , B. Wang , J. Cao , H. Wang , X. Gu , H. Huang , S. Li , X. Wang , F. Hu , M. Shao , L. Liao , T. K. Sham , Y. Liu , Z. Kang , Laser Photonics Rev. 2021, 15, 2000412.

[7] S. Zhu , Q. Meng , L. Wang , J. Zhang , Y. Song , H. Jin , K. Zhang , H. Sun , H. Wang , B. Yang , Angew. Chem., Int. Ed. 2013, 52, 3953; 10.1002/anie.201300519 23450679

[8] H. Jia , Z. Wang , T. Yuan , F. Yuan , X. Li , Y. Li , Z. Tan , L. Fan , S. Yang , Adv. Sci. 2019, 6, 1900397. 10.1002/advs.201900397 PMC6662328 31380189

[9] T. Meng , Z. Wang , T. Yuan , X. Li , Y. Li , Y. Zhang , L. Fan , Angew. Chem., Int. Ed. 2021, 60, 16343; 10.1002/anie.202103361 33960605

[10] Y. Chen , M. Zheng , Y. Xiao , H. Dong , H. Zhang , J. Zhuang , H. Hu , B. Lei , Y. Liu , Adv. Mater. 2016, 28, 312. 26568431 10.1002/adma.201503380

[11] Y. Zhan , B. Shang , M. Chen , L. Wu , Small 2019, 15, 1901161. 10.1002/smll.201901161 31045324

[12] S. Bhattacharya , R. S. Phatake , S. Nabha Barnea , N. Zerby , J. Zhu , R. Shikler , N. G. Lemcoff , R. Jelinek , ACS Nano 2019, 13, 7396. 30615415 10.1021/acsnano.8b07087

[13] J. Wang , Y. Yang , X. Liu , Mater. Adv. 2020, 1, 3122.

[14] X. Xu , Q. Chang , C. Xue , N. Li , H. Wang , J. Yang , S. Hu , J. Mater. Chem. A 2022, 10, 11712.

[15] J. Zhu , X. Bai , X. Chen , Z. Xie , Y. Zhu , G. Pan , Y. Zhai , H. Zhang , B. Dong , H. Song , Dalton Trans. 2018, 47, 3811. 29446775 10.1039/c7dt04579d

[16] J. Shao , S. Zhu , H. Liu , Y. Song , S. Tao , B. Yang , Adv. Sci. 2017, 4, 1700395. 10.1002/advs.201700395 PMC5737236 29270347

[17] X. Zhang , H. Yang , Z. Wan , T. Su , X. Zhang , J. Zhuang , B. Lei , Y. Liu , C. Hu , Adv. Opt. Mater. 2020, 8, 2000251.

[18] M. Park , Y. Jeong , H. S. Kim , W. Lee , S. H. Nam , S. Lee , H. Yoon , J. Kim , S. Yoo , S. Jeon , Adv. Funct. Mater. 2021, 31, 2102741.

[19] F. Yan , H. Zhang , J. Xu , Y. Wu , Y. Zang , J. Sun , ACS Sustainable Chem. Eng. 2021, 9, 3901.

[20] B. E. Kwak , H. J. Yoo , D. H. Kim , Adv. Opt. Mater. 2019, 7, 1900932.

[21] M. J. Islam , M. Shahjahan , K. Yuyama , V. Biju , ACS Mater. Lett. 2020, 2, 403.

[22] Z. Wang , F. Yuan , X. Li , Y. Li , H. Zhong , L. Fan , S. Yang , Adv. Mater. 2017, 29, 1702910. 10.1002/adma.201702910 28758696

[23] X. Wang , B. Wang , H. Wang , T. Zhang , H. Qi , Z. Wu , Y. Ma , H. Huang , M. Shao , Y. Liu , Y. Li , Z. Kang , Angew. Chem., Int. Ed. 2021, 60, 12585; 10.1002/anie.202103086 33754433

[24] J. Li , S. Sanz , N. Merino‐Díez , M. Vilas‐Varela , A. Garcia‐Lekue , M. Corso , D. G. de Oteyza , T. Frederiksen , D. Peña , J. I. Pascual , Nat. Commun. 2021, 12, 5538. 34545075 10.1038/s41467-021-25688-z PMC8452617

[25] C. Kütahya , P. Wang , S. Li , S. Liu , J. Li , Z. Chen , B. Strehmel , Angew. Chem., Int. Ed. 2020, 59, 3166; 10.1002/anie.201912343 PMC7027833 31724298

[26] G. Di , Z. Zhu , Q. Dai , H. Zhang , X. Shen , Y. Qiu , Y. Huang , J. Yu , D. Yin , S. Küppers , Chem. Eng. J. 2020, 379, 122296.

[27] K. Hola , M. Sudolska , S. Kalytchuk , D. Nachtigallova , A. L. Rogach , M. Otyepka , R. Zboril , ACS Nano 2017, 11, 12402. 29136460 10.1021/acsnano.7b06399

[28] S. Lu , G. Xiao , L. Sui , T. Feng , X. Yong , S. Zhu , B. Li , Z. Liu , B. Zou , M. Jin , J. S. Tse , H. Yan , B. Yang , Angew. Chem., Int. Ed. 2017, 56, 6187; 10.1002/anie.201700757 28378520

[29] K. Jiang , S. Hu , Y. Wang , Z. Li , H. Lin , Small 2020, 16, 2001909. 10.1002/smll.202001909 32597019

[30] T. Feng , S. Zhu , Q. Zeng , S. Lu , S. Tao , J. Liu , B. Yang , ACS Appl. Mater. Interfaces 2018, 10, 12262. 29164859 10.1021/acsami.7b14857

[31] S. Bhattacharya , R. S. Phatake , S. Nabha Barnea , N. Zerby , J. J. Zhu , R. Shikler , N. G. Lemcoff , R. Jelinek , ACS Nano 2019, 13, 7396. 30615415 10.1021/acsnano.8b07087

[32] X. Miao , D. Qu , D. Yang , B. Nie , Y. Zhao , H. Fan , Z. Sun , Adv. Mater. 2018, 30, 1704740. 10.1002/adma.201704740 29178388

[33] J. Luo , X. Wang , S. Li , J. Liu , Y. Guo , G. Niu , L. Yao , Y. Fu , L. Gao , Q. Dong , C. Zhao , M. Leng , F. Ma , W. Liang , L. Wang , S. Jin , J. Han , L. Zhang , J. Etheridge , J. Wang , Y. Yan , E. H. Sargent , J. Tang , Nature 2018, 563, 541. 30405238 10.1038/s41586-018-0691-0

[34] J. Chen , K. Wang , Y. Xiao , C. Cao , J. Tan , H. Wang , X. Fan , J. Yu , F. Geng , X. Zhang , C. Lee , Adv. Funct. Mater. 2021, 31, 2101647.

[35] S. Gu , M. Xia , C. Zhou , Z. Kong , M. S. Molokeev , L. Liu , W. Wong , Z. Zhou , Chem. Eng. J. 2020, 396, 125208.

