# Supplementary material for: Solid‐State Fluorescent Carbon Dots with Unprecedented Efficiency from Visible to Near‐Infrared Region
Source: Adv Sci (Weinh). 2022 Dec 3;10(4):2205788. doi: 10.1002/advs.202205788 (PMC9896040; doi:10.1002/advs.202205788)
Supplement: Supplementary file 1 — Supporting Information [file ADVS-10-2205788-s001.pdf]

ADVANCED SCIENCE
Open Access

Supporting Information

for *Adv. Sci.*, DOI 10.1002/advs.202205788

Solid-State Fluorescent Carbon Dots with Unprecedented Efficiency from Visible to Near-Infrared Region

*Bin Xu, Jie Li, Jing Zhang, Huiying Ning, Xiaoqi Fang, Jian Shen, Heng Zhou, Tianlong Jiang, Zhenhua Gao, Xiangeng Meng and Zifei Wang**

Solid-State Fluorescent Carbon Dots with Unprecedented Efficiency from Visible to Near-Infrared Region

Bin Xu[+], Jie Li[+], Jing Zhang, Huiying Ning, Xiaoqi Fang, Jian Shen, Heng Zhou, Tianlong Jiang, Zhenhua Gao, Xiangeng Meng and Zifei Wang*

B. Xu,[+] J. Li,[+] J. Zhang, H. Ning, X. Fang, J. Shen, H. Zhou, T. Jiang, Dr. Z. Gao, Prof. X. Meng, Dr. Z. Wang

School of Materials Science & Engineering, Qilu University of Technology (Shandong Academy of Sciences), Jinan, 250353, China

*E-mail: zfwang@qlu.edu.cn;

[+] These authors contributed equally to this work

Experimental Section

Materials: Perylene (98%), ethanol (99.5%), 1,3-diaminopropane (98%), sulfuric acid (95-98%), were purchased from Sinopharm Chemical Reagent Co. Ltd (Shanghai, China). All chemicals were used as received without further purification unless otherwise specified. Deionized water was used throughout this study.

Characterization Method: A JEOL JEM 2100 Transmission electron microscope (TEM) was used to examine the morphologies of CDs. Atomic force microscopy (AFM) images were taken with MultiMode V SPM (VEECO). The XRD patterns were measured by an X-ray diffraction using Cu-Kα radiation (PANalytical X'Pert Pro MPD). Optical absorption spectra were recorded on an UV-2600 spectrophotometer. The photoluminescence/phosphorescence spectra and time-resolved photoluminescence/phosphorescence decay data were obtained using a spectrometer (FLS1000) from Edinburgh Instruments. The absolute QY was obtained using Edinburgh FLS1000 fluorescence spectrophotometer equipped with a xenon arc lamp (Xe900) and an integrating sphere, respectively. The photographs were taken with camera (Nikon, D7200) under UV lamp illumination working at 365 nm (UV lamp: SPECTROLINE, ENF-280C/FBE, 8W). The FTIR spectra were measured using a Nicolet 380 spectrograph. The XPS spectra were measured with an ESCALab220i-XL electron spectrometer from VG Scientific using 300 W Al Kα radiation. The rheological property was measured using a 40 mm parallel plate with a gap of 1000 μm on a hybrid rheometer (Discovery HR-2, TA Instrument).

Synthesis of solid-state CDs: The highly efficient Y-CDs, R-CDs, DR-CDs and NIR-CDs were synthesized by solvothermal method. The perylene (0.15 g), 1,3-diaminopropane (10 mL) and sulfuric acid (40, 50, 70, 80 μl) were dissolved in ethanol (10 mL), stirred for 15 min, then the solution was transferred to a 50 ml poly (tetrafluoroethylene)-lined autoclave and heated at 200 °C for 6, 8, 10, and 15 h. After the reaction, the reactors were cooled to room temperature naturally. Subsequently, the brown solution was centrifuged at 10000 rad/min (10 min) for 3 times to remove the transparent liquid. Finally, the solid Y-CDs, R-CDs, DR-CDs and NIR-CDs powders were obtained by dialyzing with a dialysis membrane with a molecular weight cutoff (MWCO: 1000Da) for 3 days and oven drying, respectively.

Preparation of white LEDs: A blue chip centered at 450 nm was used for the fabrication of white LEDs. 0.1 g of Y-CDs, 0.1 g of Y-CDs/R-CDs (mass ratio of 2.8:1.3), and 0.1 g of Y-CDs/R-CDs/NR-CDs (mass ratio of 3.1:1.5:0.6) powders were added into 0.4 g epoxy resin, respectively, and constantly stirred to form a homogeneous and viscous mixture. Subsequently, part of the mixture was coated on the center of the blue chip and transferred to an electric thermostatic blast oven for curing at 60 °C for 45 min to obtain three white LED (white LED-1, white LED-2 and white LED-3) devices.

Preparation of plant growth LED: A mixture of 0.5g epoxy resin, 0.21 g DR-CDs and 0.18 g NIR-CDs phosphor was coated on a blue chip (λ_{em} = 450 nm), and transferred to the electrothermal thermostat blower and cured at 60 °C for 45 min to obtain the plant growth LED device.

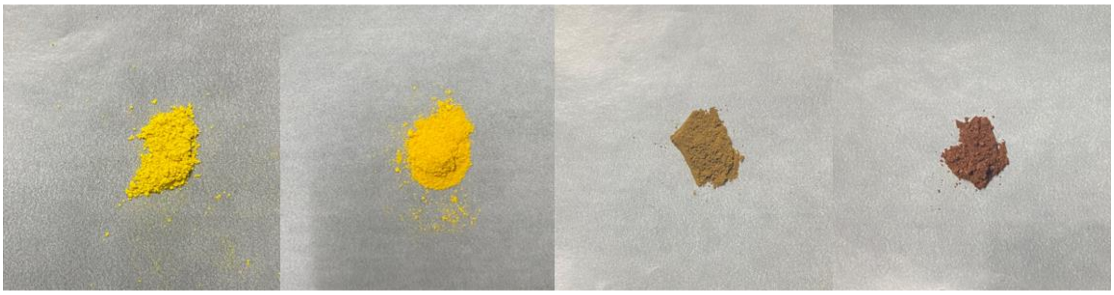

Figure S1 Photographs for Y-CDs, R-CDs, DR-CDs, and NIR-CDs under sunlight.

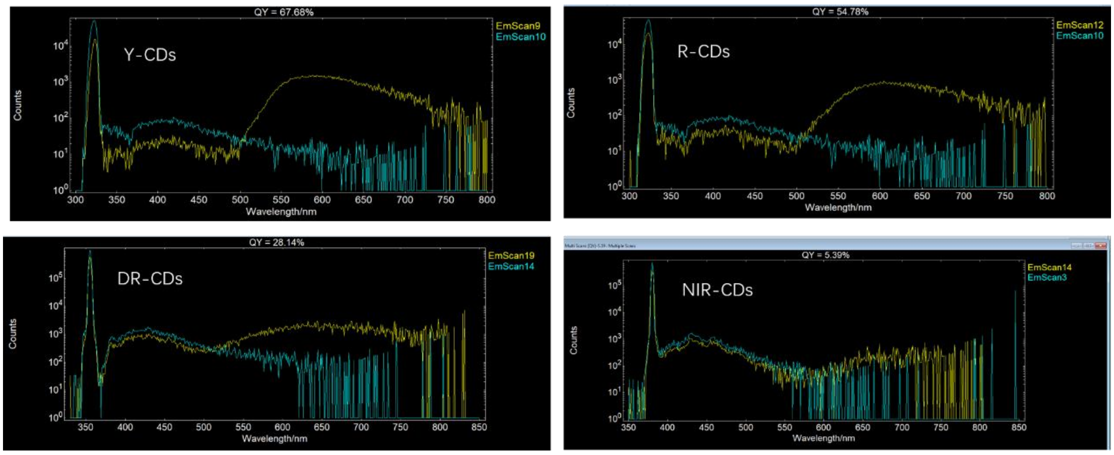

Figure S2 The quantum yield test data of Y-CDs, R-CDs, DR-CDs, and NIR-CDs.

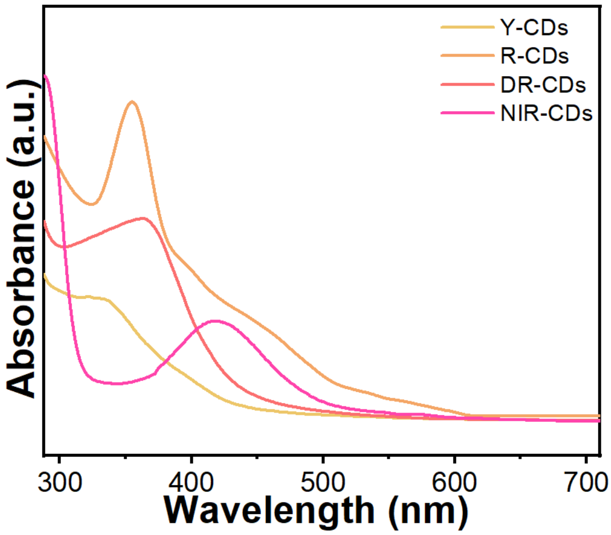

Figure S3 UV–vis absorption spectra of Y-CDs, R-CDs, DR-CDs, and NIR-CDs aqueous dispersion, respectively.

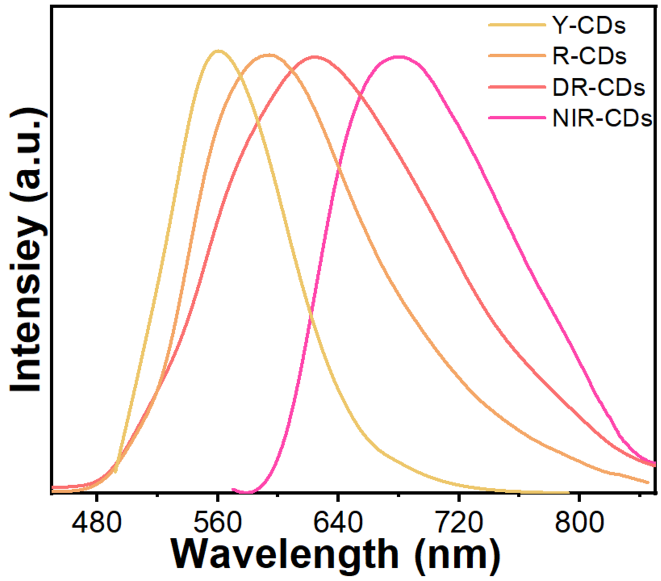

Figure S4 PL spectra of Y-CDs, R-CDs, DR-CDs, and NIR-CDs aqueous dispersion, respectively.

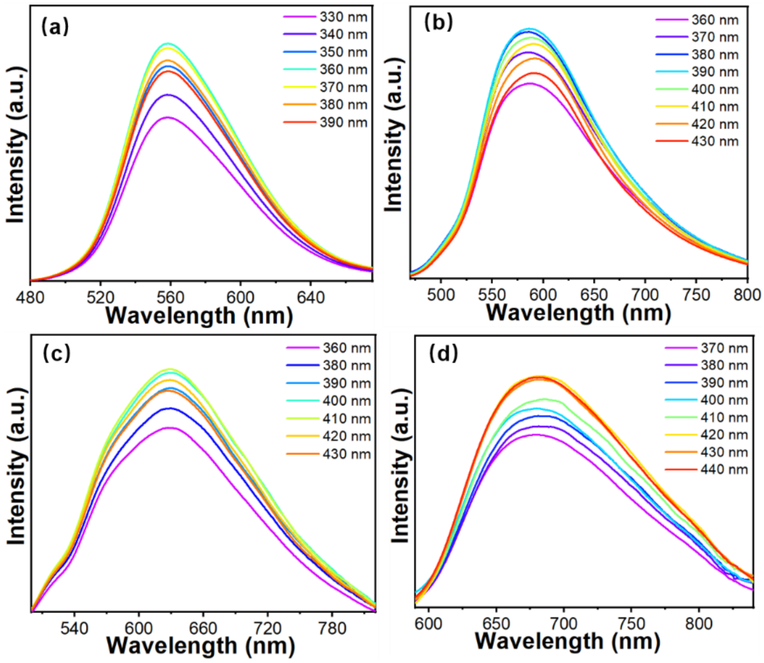

Figure S5 Evolution of the PL spectra with the excitation wavelength for the Y-CDs (a), R-CDs (b), DR-CDs (c), and NIR-CDs (d) aqueous dispersion.

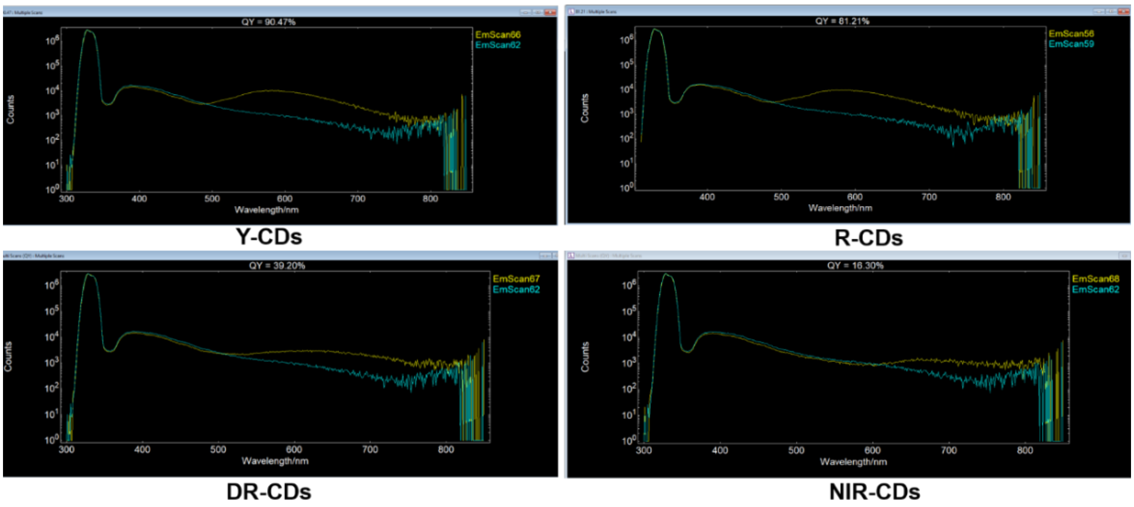

Figure S6 The quantum yield test data of Y-CDs, R-CDs, DR-CDs, and NIR-CDs aqueous dispersion.

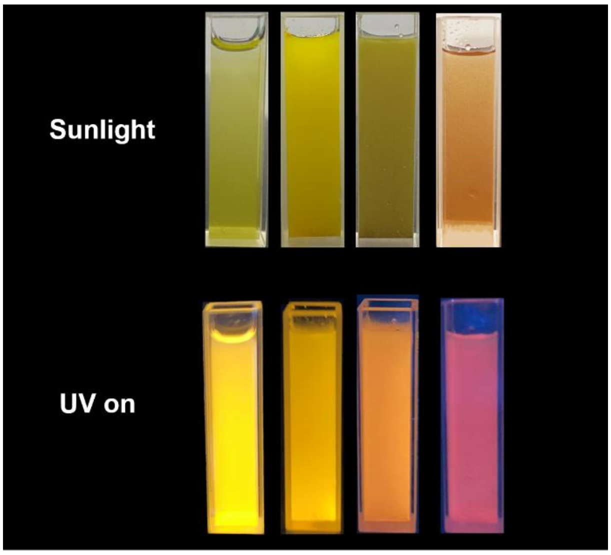

Figure S7 Photographs of Y-CDs, R-CDs, DR-CDs, and NIR-CDs aqueous dispersion under sunlight (above) and 365 nm UV lamp illumination (below).

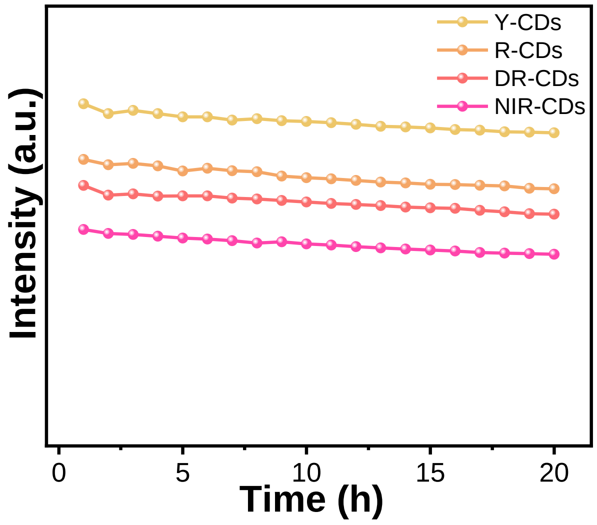

Figure S8 Photostability of Y-CDs, R-CDs, DR-CDs, and NIR-CDs under continuous illumination with an UV (365 nm) beam for 20 h.

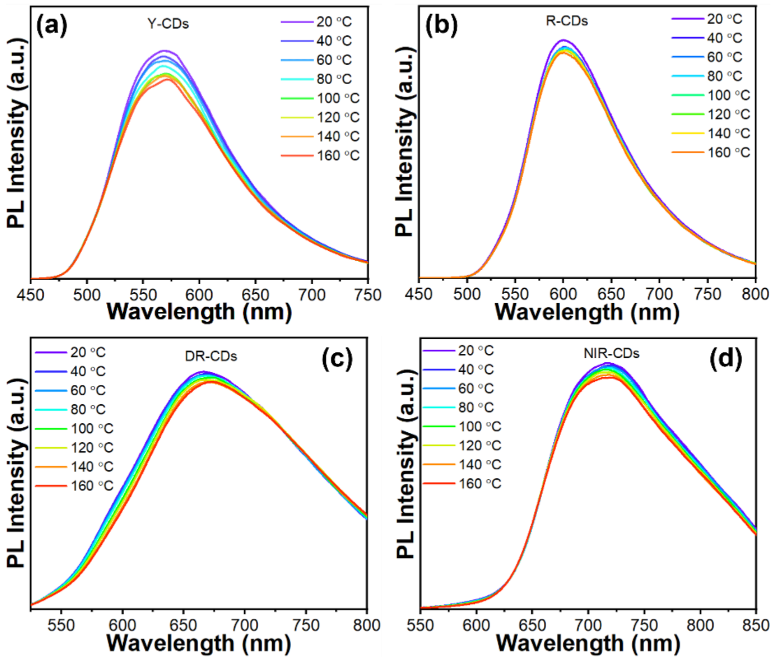

Figure S9 PL spectra of (a) Y-CDs, (b) R-CDs, (c) DR-CDs, and (d) NIR-CDs at different temperatures from 20 to 160 °C, under excitation at 365nm.

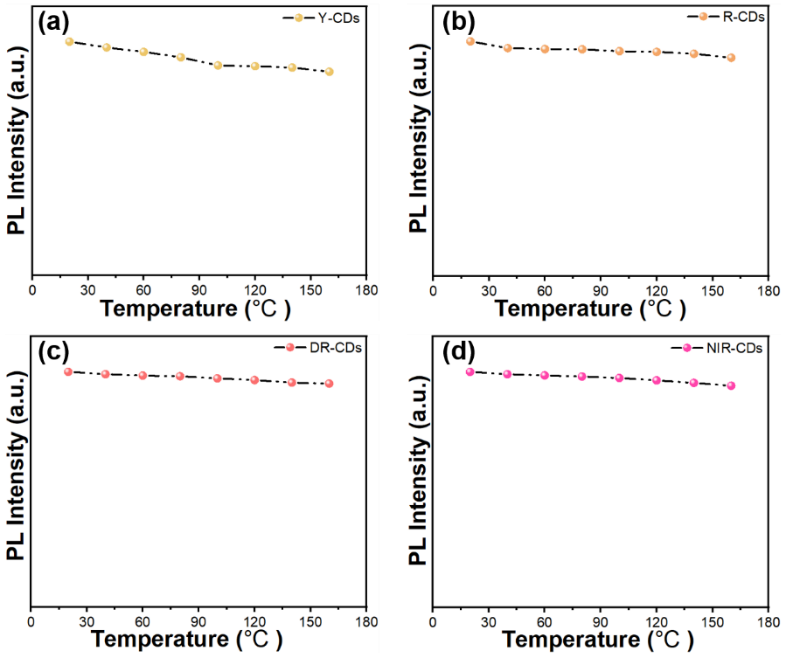

Figure S10 The PL intensity of (a) Y-CDs, (b) R-CDs, (c) DR-CDs, and (d) NIR-CDs at different temperatures.

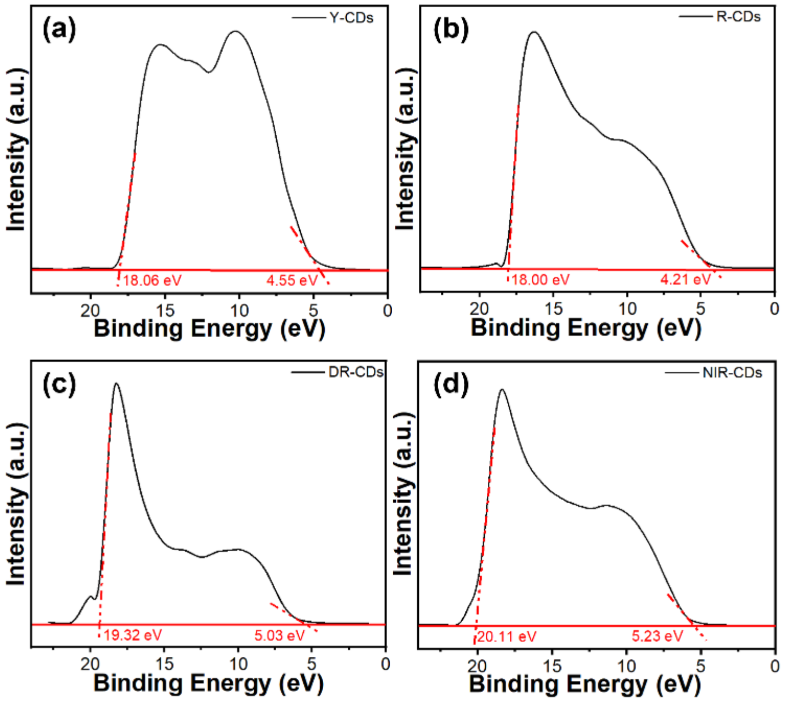

Figure S11 UPS results of (a) Y-CDs, (b) R-CDs, (c) DR-CDs, and (d) NIR-CDs. The left point of intersection is end edge and the right one is fermi edge.

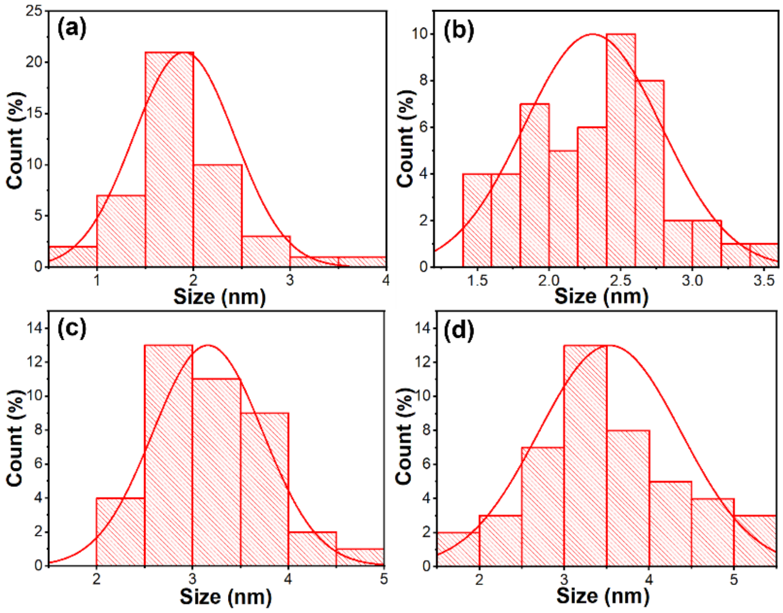

Figure S12 The size distributions of (a) Y-CDs, (b) R-CDs, (c) DR-CDs, and (c) NIR-CDs.

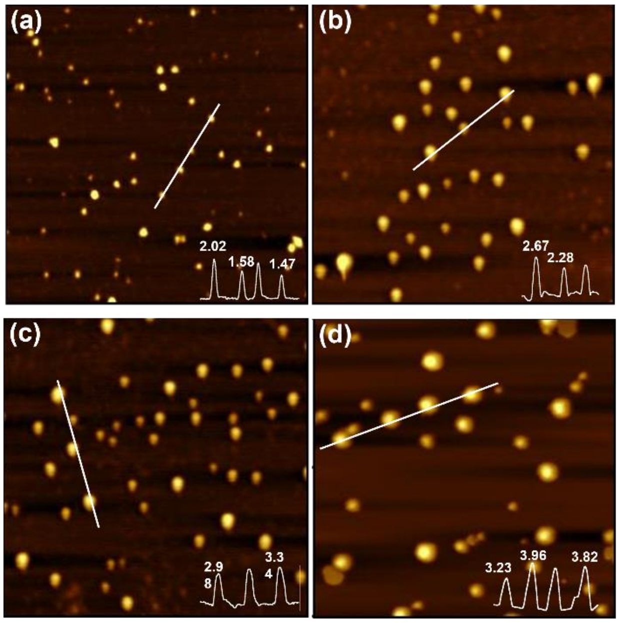

Figure S13 AFM images and corresponding height profiles of the (a) Y-CDs, (b) R-CDs, (c) DR-CDs, and (c) NIR-CDs.

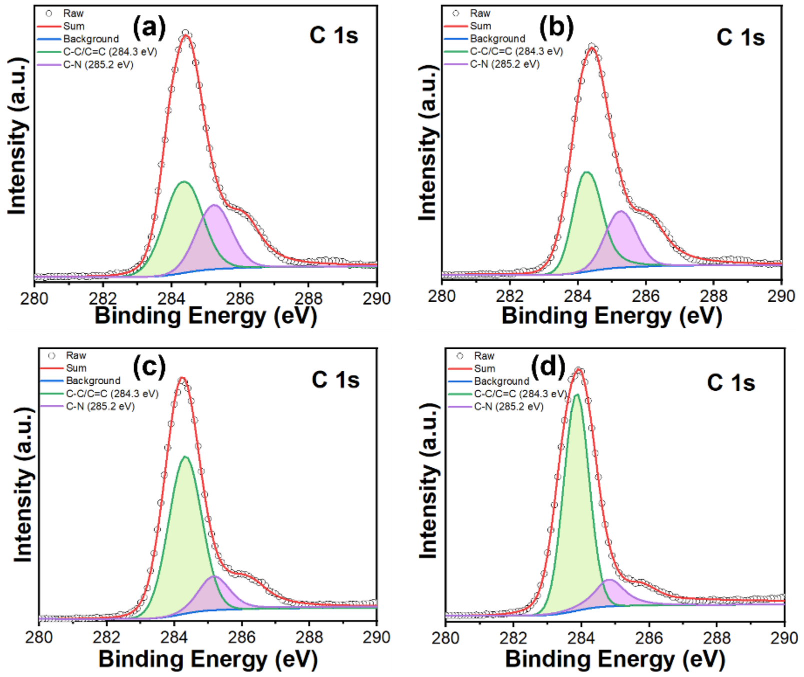

Figure S14 High-resolution XPS C1s spectra of (a) Y-CDs, (b) R-CDs, (c) DR-CDs, and (d) NIR-CDs.

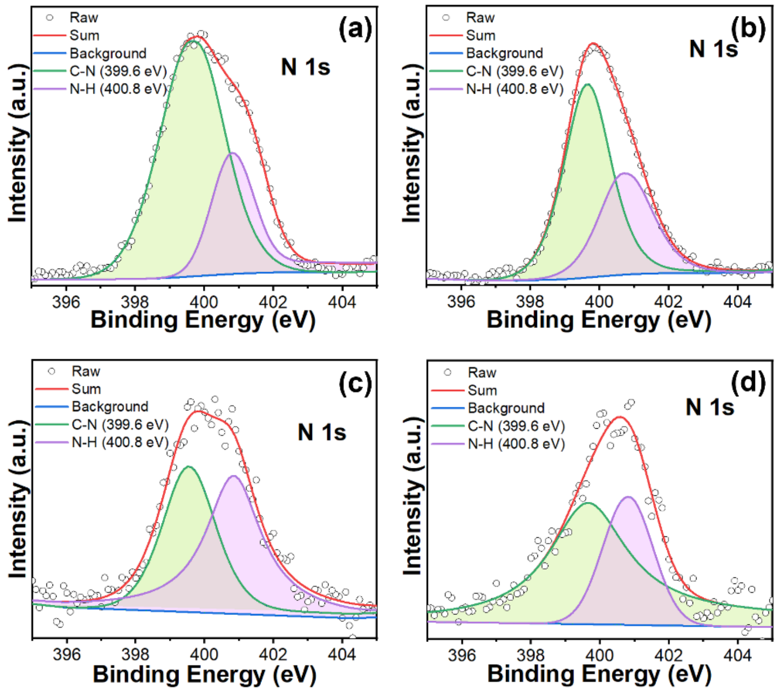

Figure S15 High-resolution XPS N1s spectra of (a) Y-CDs, (b) R-CDs, (c) DR-CDs, and (d) NIR-CDs.

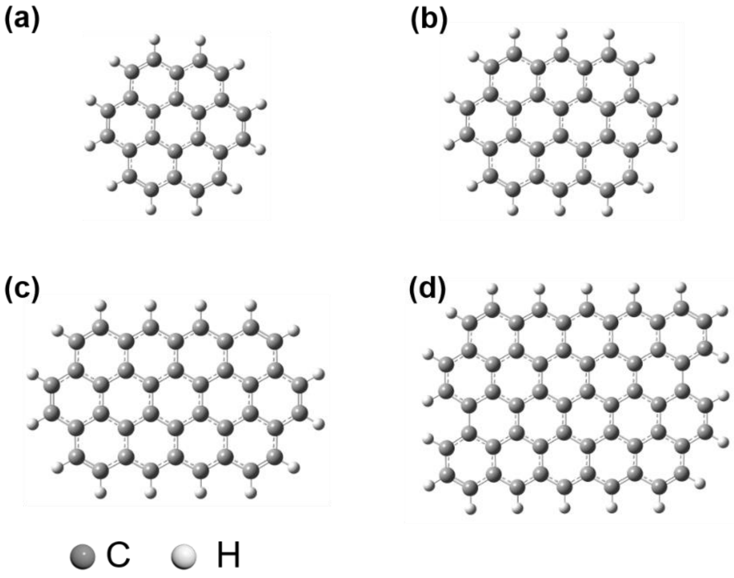

Figure S16 HOMO and LUMO states of the established model by increasing the aromatic rings (a-d).

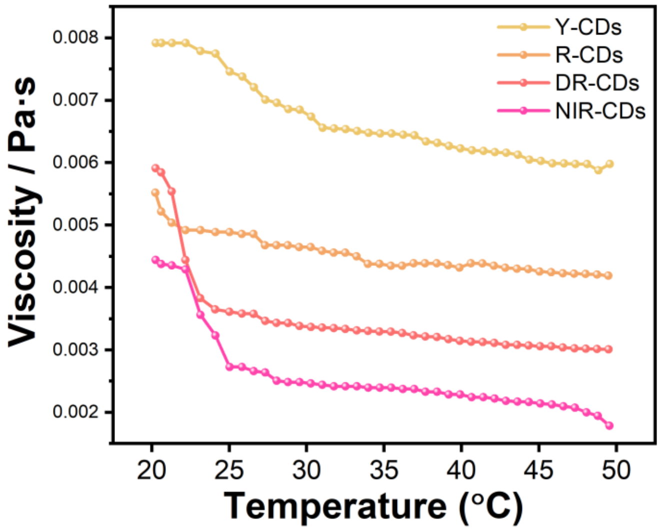

Figure S17 Effect of temperature on the viscosity of Y-CDs, R-CDs, DR-CDs, and NIR-CDs aqueous dispersion.

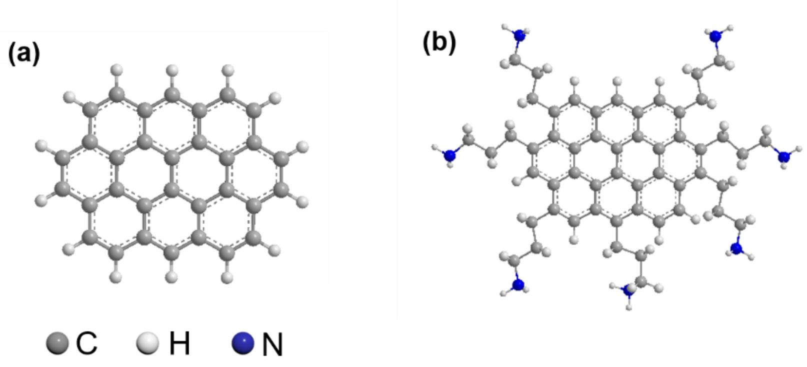

Figure S18 Molecular dynamics structure models of bare CDs (a) and PF-CDs (b).

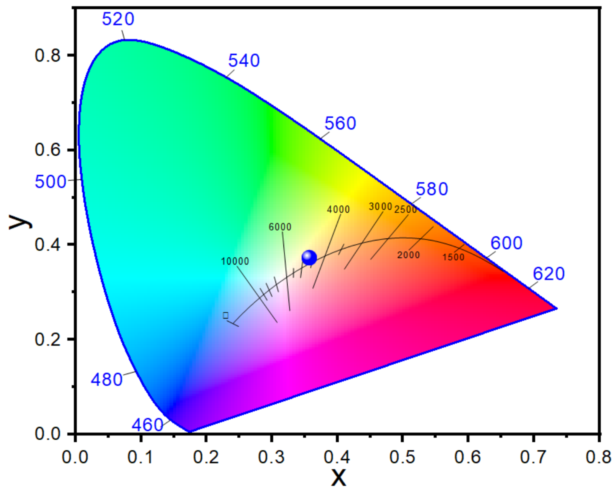

Figure S19 CIE color coordinates of the white LED lamp with excitation at 450 nm.

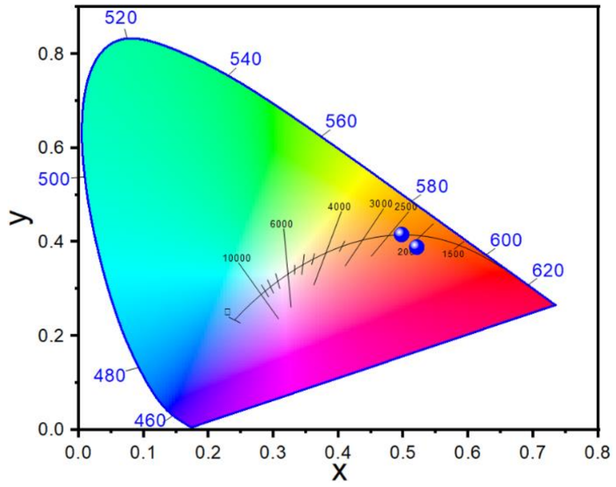

Figure S20 CIE color coordinates of the warm white LED lamps with excitation at 450 nm.

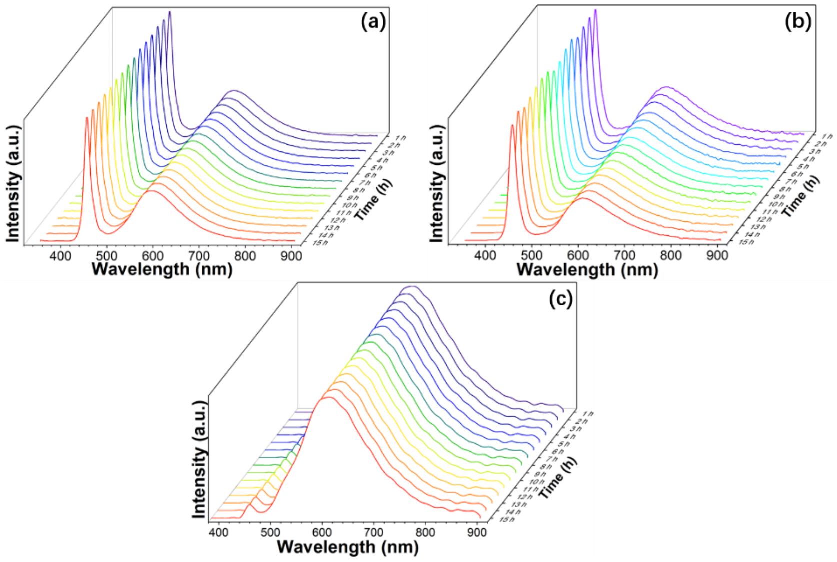

Figure S21 PL spectra of (a) white and (b, c) warm white LEDs at different operating time intervals.

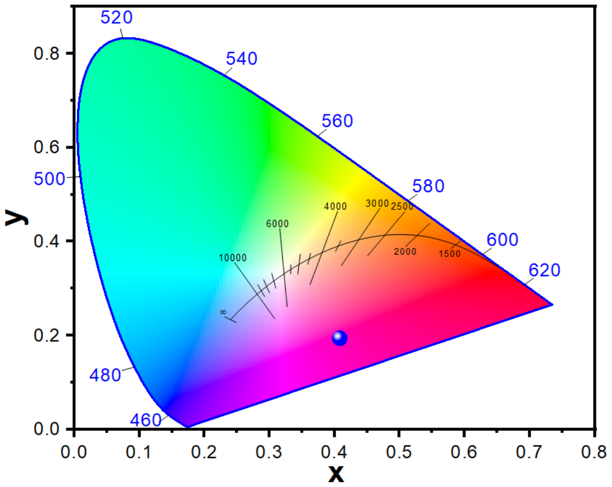

Figure S22 CIE color coordinates of the plant growth LED lamp.

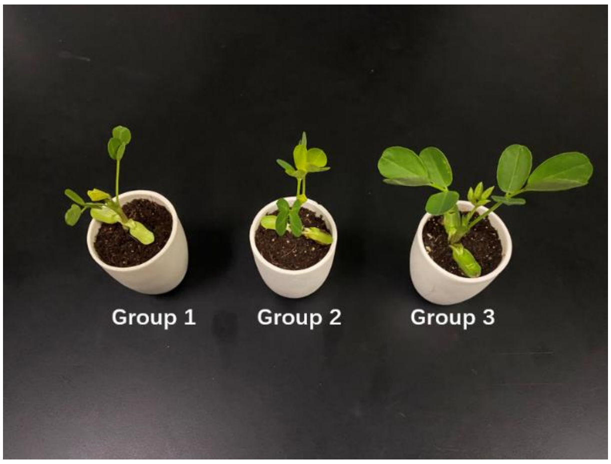

Figure S23 Digital photograph of peanut cultivation irradiated by different lighting sources after 7 days.

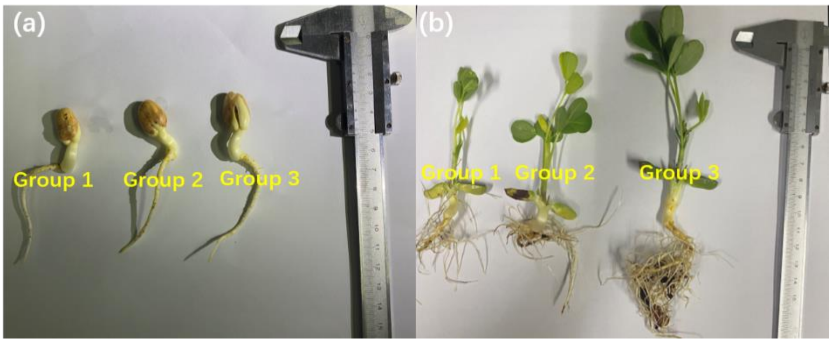

Figure S24 (a) The height of peanuts (group 1-3) when they first sprout. (b) The height of peanuts (group 1-3) cultivation irradiated by different lighting sources after 7 days.

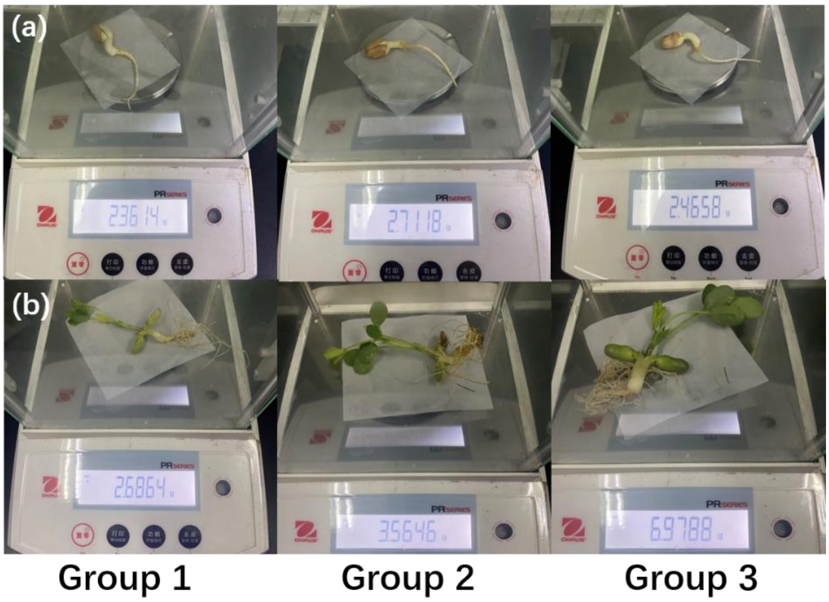

Figure S25 (a) The weight of peanuts (group 1-3) when they first sprout; (b) The weight of peanuts (group 1-3) cultivation irradiated by different lighting sources after 7 days.

Table S1 Comparision of FL emission wavelengths and QYs with other solid-state CD materials.

Sample	FL emission wavelength	QYs	Reference
**C-dots**	606-653 nm	8.1-25.4 %	[1]
**R-CDs**	620 nm	12.7 %	[2]
**PCDs**	575-625 nm	5.54-8.5%	[3]
**CDs**	550-586 nm	6.78 %	[4]
**Y-CD**	491-557 nm	23.7 %	[5]
**CDs@MOF**	450-590 nm	19.5 %	[6]
**R/G/B-SBF-CQRs**	474-630 nm	30-46 %	[7]
**Y-/R-/DR-/NIR-CDs**	570-721 nm	5.39-67.6%	This work

Table S2 Fluorescence-decay lifetimes and fitting parameters of Y-CDs, R-CDs, DR-CDs, and NIR-CDs.

Sample	τ_1 (ns)	B_1 (%)	τ_2 (ns)	B_2 (%)	τ_{avg} (ns)	x^2
**Y-CDs**	1.02	35.34	3.07	64.66	2.35	1.033
**R-CDs**	0.83	34.65	1.14	65.35	1.03	1.082
**DR-CDs**	0.79	30.91	0.98	69.09	0.92	1.104
**NIR-CDs**	0.62	27.27	0.71	72.73	0.68	1.154

Table S3 Relative contents of different functional groups in the Y-CDs, R-CDs, DR-CDs, and NIR-CDs, respectively.

**Sample**	C 1s (%)		N 1s (%)	
	C-C/C=C	C-N	C-N	N-H
**Y-CDs**	64.33	35.67	76.41	23.59
**R-CDs**	72.63	27.37	70.29	29.71
**DR-CDs**	79.38	20.62	65.35	34.65
**NIR-CDs**	85.21	14.79	60.33	39.67

Table S4 Relative contents of different element in the Y-CDs, R-CDs, DR-CDs, and NIR-CDs, respectively.

**Sample**	C 1s (%)	N 1s (%)
**Y-CDs**	68.45	31.55
**R-CDs**	72.62	27.38
**DR-CDs**	77.53	22.47
**NIR-CDs**	84.67	15.33

Table S5 Comparison of performance parameters of various WLEDs.

Materials	CRI	CCT (K)	Luminous efficacy (lm/W)	References
**$CsPb_{0.64}Sn_{0.36}Br_3$ QDs glass**	74.2	3128-6119	29. 06	[8]
**A-CDs**	–	3791	31.6	[9]
**F-embedded CDs**	95	5232	–	[10]
**AC-CD**	92	–	30.5	[11]
**B-CQD, G-CQD, and R-CQD**	93	3774	31.3	[12]
**CDs**	94	5612	–	[13]
**Commercial WLED**	74	5375	89.9	[14]
**Y-, R-, DR-, and NIR-CDs**	75.9	1882-5019	54.6-87.5	This work

Reference

[1] C. Ji, Q. Han, Y. Zhou, J. Wu, W. Shi, L. Gao, R. M. Leblanc, Z. Peng, *Carbon* **2022**, *192*, 198.

[2] X. Zhang, H. Yang, Z. Wan, T. Su, X. Zhang, J. Zhuang, B. Lei, Y. Liu, C. Hu, *Adv. Opt. Mater.* **2020**, *8*, 2000251.

[3] M. Park, Y. Jeong, H. S. Kim, W. Lee, S. H. Nam, S. Lee, H. Yoon, J. Kim, S. Yoo, S. Jeon, *Adv. Funct. Mater.* **2021**, *31*, 2102741.

[4] S. Lu, G. Xiao, L. Sui, T. Feng, X. Yong, S. Zhu, B. Li, Z. Liu, B. Zou, M. Jin, J. S. Tse, H. Yan, B. Yang, *Angew Chem Int Ed*. **2017**, *56*, 6187.

[5] J. Shao, S. Zhu, H. Liu, Y. Song, S. Tao, B. Yang, *Adv. Sci*. **2017**, *4*, 1700395.

[6] Y. Ma, X. Zhang, J. Bai, K. Huang, L. Ren, *Chem. Eng. J.* **2019**, *374*, 787.

[7] T. Meng, Z. Wang, T. Yuan, X. Li, Y. Li, Y. Zhang, L. Fan, *Angew. Chem. Int. Ed.* 2021, *60*, 16343.

[8] S. Liu, G. Shao, L. Ding, J. Liu, W. Xiang, X. Liang, *Chem. Eng. J.* **2019**, *361*, 937.

[9] J. Zhu, J. Hu, Q. Hu, X. Zhang, E.V. Ushakova, K. Liu, S. Wang, X. Chen, C. Shan, A.L. Rogach, X. Bai, *Small* **2022**, *18*, 2105415.

[10] R. Kumari, A. Kumar, N.K. Mishra, S.K. Sahu, *Langmuir* **2022**, *38*, 9389.

[11] T. Zhang, F. Zhao, L. Li, B. Qi, D. Zhu, J. Lü, C. Lü, *ACS Appl. Mater. Interfaces* **2018**, *10*, 19796.

[12] Z. Wang, F. Yuan, X. Li, Y. Li, H. Zhong, L. Fan, S. Yang, *Adv Mater*. **2017**, *29*, 1702910.

[13] Y. Zheng, K. Arkin, J. Hao, S. Zhang, W. Guan, L. Wang, Y. Guo, Q. Shang, *Adv. Opt. Mater*. **2021**, *9*, 2100688.

[14] T. Nguyen, T. Luu, D. Nguyen, T. Duong, *J. Electron. Mater.* **2021**, *50,* 1827.
